# O04 - Effects of 5-lipoxygenase pathway inhibition on rhinovirus-associated bronchial epithelial inflammation

**DOI:** 10.1186/2045-7022-4-S1-O4

**Published:** 2014-03-14

**Authors:** Irini Spyridaki, Chrysanthi Skevaki, Aikaterini Trochoutsou, Styliani Taka, Nikolaos Papadopoulos

**Affiliations:** 1 Allergy Research Laboratories, Second Department of Pediatrics, University of Athens, Athens, Greece

## Background

Human bronchial epithelial cells produce a variety of inflammatory mediators upon exposure to rhinovirus (RV), a major precipitant of asthma exacerbations. We hypothesized that anti-leukotriene (LT) treatment of epithelial cells with or without exposure to supernatants of RV-infected peripheral blood mononuclear cells (PBMCs) may inhibit RV-induced up-regulation of inflammatory cytokines.

## Methods

PBMCs were isolated from a non-atopic, non-asthmatic donor and exposed to 1MOI of RV-1B, and supernatants were harvested at 48h post-infection. Subsequently, BEAS-2B, a bronchial epithelial cell line, was infected with RV, with or without conditioning with PBMC supernatants. Treatment with anti-LT agents was performed either on both PBMCs and BEAS-2B or at the bronchial epithelial level only, with varying concentrations of montelukast or MK-886. We evaluated the concentration of inflammatory cytokines (IL-8, RANTES, IL-11, IL-6 and IP-10) in culture supernatants at 48 hours after infection of BEAS-2B cells with the use of commercially available immunoassays.

## Results

Treatment of PBMCs and BEAS-2B with montelukast at concentrations of 10^-4^M to 10^-6^M, and with MK-886 at a concentration of 10^-4^M significantly inhibited release of IL-8 by RV-infected BEAS-2B. RANTES, IL-6 and IP-10 release was inhibited at all concentrations tested by both drugs, while IL-11 release was inhibited only after treatment with montelukast. Treatment of BEAS-2B cells with montelukast or with MK-886 at a concentration of 10^-6^M inhibited release of all cytokines measured, irrespective of exposure to conditioned media of RV-infected PBMCs, while 10^-9^M montelukast inhibited the release of IL-8, IL-11 and IL-6, and 10^-9^M MK-886 suppressed the release of IL-8 and RANTES.

## Conclusion

Our results show that anti-LT treatment of RV-infected bronchial epithelial cells, with or without exposure to conditioned media of RV-infected PBMCs suppresses epithelial RV-mediated inflammatory cytokine production. Our observations may represent an indirect mode of action of anti-leukotriene medication in virus-induced asthma.

